# Longitudinal evaluation of myocardial glucose metabolism and contractile function in obese type 2 diabetic db/db mice using small-animal dynamic ^18^F-FDG PET and echocardiography

**DOI:** 10.18632/oncotarget.21202

**Published:** 2017-09-23

**Authors:** Kuan-Yin Ko, Yen-Wen Wu, Cheng-Wei Liu, Mei-Fang Cheng, Ruoh-Fang Yen, Wei-Shiung Yang

**Affiliations:** ^1^ Department of Nuclear Medicine, National Taiwan University Hospital, Yunlin Branch, Yunlin County, Taiwan; ^2^ Department of Nuclear Medicine, National Taiwan University Hospital and National Taiwan University, College of Medicine, Taipei, Taiwan; ^3^ Department of Internal Medicine, National Taiwan University Hospital and National Taiwan University, College of Medicine, Taipei, Taiwan; ^4^ National Yang-Ming University School of Medicine, Taipei, Taiwan; ^5^ Cardiology Division of Cardiovascular Medical Center, Far Eastern Memorial Hospital, New Taipei City, Taiwan; ^6^ Department of Nuclear Medicine, Far Eastern Memorial Hospital, New Taipei City, Taiwan; ^7^ Department of Internal Medicine, Tri-Service General Hospital, Songshan Branch, National Defense Medical Center, Taipei, Taiwan; ^8^ Graduate Institute of Clinical Medicine, College of Medicine, National Taiwan University, Taipei, Taiwan; ^9^ Institute of Occupational Medicine and Industrial Hygiene, National Taiwan University, Taipei, Taiwan; ^10^ Department of Medicine and Graduate Institute of Medical Genomics & Proteomics, College of Medicine, National Taiwan University, Taipei, Taiwan; ^11^ R & D Branch Office, College of Medicine, National Taiwan University, Taipei, Taiwan

**Keywords:** type 2 diabetes mellitus (DM), db/db mice, small animal imaging, positron emission tomography (PET), ^18^F-fluorodeoxyglucose (^18^F-FDG)

## Abstract

The aim was to evaluate sequential changes of myocardial glucose utilization and LV systolic function in db/db mice.

Eight db/db and eight wild-type mice underwent plasma substrate analysis and dynamic ^18^F-FDG PET at week 8 (W8), W10, W12, W14, and W16. ^18^F-FDG uptake constant Ki and the rate of myocardial glucose uptake (MRGlu) were derived via Patlak graphic analysis. Another 8 db/db and 8 wild-type mice received echocardiography at W8, W12, and W16 and LV structure and function were measured.

The db/db mice showed increased weights and glucose levels as they aged. The index of homeostasis model assessment-estimated insulin resistance, insulin, and free fatty acid concentrations were higher in db/db mice compared with wild-type. MRGlu of db/db mice across all time points was markedly higher than that of wild-type. An age-dependent elevation of MRGlu was observed in db/db mice. Ki and MRGlu of db/db mice showed negative correlation with triglyceride levels. When two groups were pooled together, Ki and MRGlu were significantly proportional to glucose levels. No significant difference in LV structure and function was noted between db/db and control mice.

In conclusion, we demonstrated altered myocardial glucose utilization preceding the onset of LV systolic dysfunction in db/db mice.

## INTRODUCTION

Cardiovascular disease has been recognized as a major complication of type 2 diabetes mellitus (DM). Accumulating data have shown that DM results in cardiac functional and structural changes. The changes are independent of hypertension and coronary artery disease, thereby supporting the existence of diabetic cardiomyopathy [1]. It is important to note that diabetes-associated changes are amplified by the existence of co-morbidities which can augment development of left ventricular (LV) hypertrophy, increase susceptibility of the heart to ischemic injury, and increase the overall likelihood of developing heart failure [2]. Although several mechanisms, including myocardial fibrosis, small vessel disease, autonomic dysfunction and insulin resistance, have been put forth to explain diabetic cardiomyopathy, growing evidence suggests that the condition is partly a consequence of severe alteration in myocardial energy metabolism [3–6] and the diabetic heart primarily relied on fatty acid utilization for energy production.

Positron emission tomography (PET) has been used to quantify myocardial glucose use noninvasively in humans [7–9]. Small-animal PET systems have been introduced, allowing for high-resolution preclinical small-rodent PET imaging [10, 11]. Compared with qualitative interpretation in static imaging, dynamic ^18^F-FDG-PET imaging offers more detailed information on myocardial metabolism, including absolute quantification of the myocardial metabolic rate of glucose utilization. The latter is achieved through analysis by compartmental modeling.

Several applications of dynamic small animal ^18^F-FDG PET have been proposed. Yamane et al. [12] have developed a novel assay adapted to available small-animal PET for reliable quantification of myocardial tracer kinetics. Dynamic small-animal PET has helped to clarify mechanisms responsible for the metabolic alterations that occur in various diseases and pathologic processes, including diabetes [13, 14] and LV hypertrophy [15, 16]. The cited studies highlight advances in small-animal PET technology that enable quantification of metabolic parameters and exemplify the translational capabilities of metabolic PET.

Consequently, the aim of this study was to evaluate non-invasively the time course of myocardial glucose utilization and LV systolic function in C57BL/KSJ-lepr^db^/lepr^db^ (*db/db*) mice, which has a mutation in the leptin receptor. Lepr^db^ mutation is a recessive mutation on chromosome 4 that occurred in C57BLKS/J inbred strain in 1966. The Lepr^db^ mutation was subsequently transferred to the C57BLKS/J inbred strain by backcrossing [17, 18]. It was a well-established animal model displaying type 2 DM characteristics, such as hyperglycemia, insulin resistance, obesity due to hyperphagia, and reduced energy expenditure [19–21]. Evidence of diabetic cardiomyopathy has also been reported in db/db mice. Systolic and diastolic dysfunction were detected by echocardiography [22]. With the use of ex vivo perfused hearts from db/db mice, augmented LV end-diastolic pressure and reduced cardiac output and cardiac power have also been observed [23]. Early alteration of substrate metabolism might develop without contractile dysfunction [23, 24]. To our knowledge, there was no longitudinal in-vivo evaluation of myocardial metabolic alterations of db/db mice using dynamic ^18^F-FDG PET. We hypothesize that altered myocardial glucose utilization occurs in the early stage of diabetic cardiomyopathy, prior to LV structure change and contractile dysfunction.

## RESULTS

**Figure 1:**
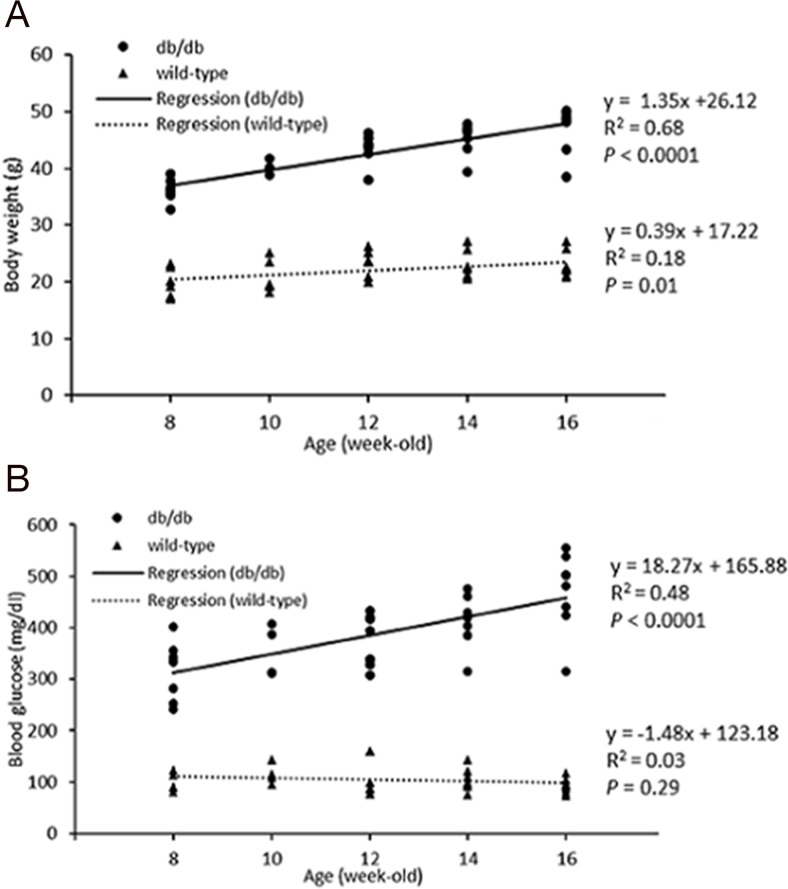
Body weight **(A)** and blood glucose **(B)** of wild type and db/db mice at 8, 10, 12, 14, and 16 weeks of ages. Body weight and glucose levels in db/db demonstrated moderate age-dependent increases. Body weight, not glucose, in wild type mice increased only minimally with age. *P* value of less than 0.05 was considered significant.

The fasting glucose levels of db/db mice were significantly greater than those of age-matched controls (all *P*<0.05). In the db/db group, body weight increased as animals got older, but wild type mice showed only a mild age-dependent increase in body weight (Table 1, 2). Body weight and blood glucose were positively correlated with age in db/db mice (Figure 1); there was no correlation of body weight and glucose levels in the control group.

**Table 1:** Clinical characteristics, plasma substrate levels, HOMA-IR index and estimated metabolic glucose utilization using FDG PET of wild type and db/db animal models

Mice group (n)	Weight (g)	TG (nmol/μl)	FFA (nmol/μl)	HOMA-IR			MRGlu (umol/min/100g)		Isolated Heart		
				Glucose (mg/dl)	Insulin (μg/l)	Index	Max	Mean	LVW (g) (n=4/group)	HW (g) (n=4/group)	HW/BW (% (n=4/group)
**W8**											
wild-type (4)	19.99 ± 2.59	0.57 ± 0.15	0.36 ± 0.07	98.50 ± 16.62	0.06 ± 0.01	0.45 ± 0.01	14.15 ± 5.76	2.43 ± 1.41			
db/db (7)	36.17 ± 1.84^*^	0.69 ± 0.23	4.27 ± 2.14^*^	318.25 ± 54.84^*^	0.08 ± 0.01	1.89 ± 0.07^*^	79.64 ± 27.10^*^	35.95 ± 19.70^*^			
**W10**											
wild-type (6)	20.85 ± 2.84	0.54 ± 1.68	0.38 ± 0.04	120.33 ± 19.33	0.06 ± 0.01	0.54 ± 0.02	24.00 ± 7.32	7.25 ± 3.71			
db/db (4)	40.25 ± 1.17^*†^	0.79 ± 0.10	2.31 ± 2.47^*^	354.75 ± 50.10^*^	0.11 ± 0.01	2.84 ± 0.03^*^	87.24 ± 25.61^*^	45.12 ± 31.40^*^			
**W12**											
wild-type (4)	23.26 ± 2.42	1.20 ± 0.31	0.44 ± 0.01	112.00 ± 38.20	0.09 ± 0.04	0.72 ± 0.11	11.03 ± 6.21	0.88 ± 0.43			
db/db (5)	43.48 ± 2.47^*†^	2.10 ± 0.25	1.47 ± 0.51^*^	372.12 ± 49.25^*^	0.11 ± 0.02	2.97 ± 0.08^*†^	77.69 ± 34.88^*^	19.28 ± 15.96^*^			
**W14**											
wild-type (7)	22.86 ± 2.36	0.81 ± 0.34	0.46 ± 0.10	104.12 ± 20.53	0.05 ± 0.01	0.44 ± 0.02	12.27 ± 6.83	3.66 ± 1.85			
db/db (5)	45.38 ± 2.76^*†^	0.61 ± 0.23	2.78 ± 3.05^*^	414.25 ± 49.10^*†^	0.08 ± 0.02	2.29 ± 0.06^*†^	165.80 ± 87.93^*†^	76.66 ± 65.46^*†^			
**W16**											
wild-type (7)	23.09 ± 2.23	0.80 ± 0.28	0.46 ± 0.05	93.50 ± 14.00	0.06 ± 0.01	0.43 ± 0.01	16.58 ± 5.81	3.63 ± 3.60	0.08 ± 0.04	0.11 ± 0.05	0.48 ± 0.16^‡^
db/db (7)	47.03 ± 4.03^*†^	0.55 ± 0.20	1.15 ± 0.42^*^	469.75 ± 76.86^*†^	0.07 ± 0.01	2.35 ±0.06^*†^	129.13 ± 48.49^*†^	71.90 ± 40.37^*†^	0.08 ± 0.03	0.12 ± 0.03	0.26 ± 0.06

TG, triglyceride. FFA, free fatty acid. HOMA-IR, homeostasis model assessment-estimated insulin resistance. MRGlu, rate of myocardial glucose uptake. LVW, left ventricular weight. HW, heart weight. BW, body weight.

^*^Significantly higher than wild-type group. ^†^Significantly higher than baseline W8 values. ^‡^Significantly higher than db/db group. *P*<0.05 was considered significant.

The echocardiographic measurements are summarized in Table 2. Comparing db/db and their age-matched controls, there was no significant difference in LV volumetric data, including wall thickness, systolic and diastolic dimensions, and fractional shortening and LVEF. Neither db/db nor control mice displayed a significant change in LV parameters with age. In the end of the experiments (16~17 weeks of age), there were no significant differences in LV weight (LVW) and heart weight (HW) between the two groups (Table 1, 2); while db/db mice had significantly higher body weight, resulting in a significantly lower HW/BW ratio.

Circulating substrate values, including glucose, FFA, TG, insulin, and the HOMA-IR index are summarized in Table 1. Glucose, FFA levels, and HOMA-IR index of the db/db group were significantly higher compared with age-matched controls. When all time points were combined between W8 to W16, insulin levels in db/db mice were higher than in wild-type mice. Between 8 to 12 weeks, db/db mice tended to have higher TG levels, which declined at ages 14 and 16 weeks, with no statistical differences.

**Table 2:** Clinical and echocardiographic characteristics in wild type and db/db mice

	Wild-type (n)			db/db (n)		
Parameters	W8 (8)	W12 (7)	W16 (7)	W8 (7)	W12 (7)	W16 (7)
**UCG**						
IVS, mm	0.68 ± 0.15	0.64 ± 0.08	0.60 ± 0.10	0.75 ± 0.93	0.68 ± 0.05	0.68 ± 0. 06
PW, mm	0.72 ± 0.73	0.70 ± 0.11	0.63 ± 0.11	0.62 ± 0.11	0.69 ± 0.07	0.70 ± 0.08
EDD, mm	3.94 ± 0.42	4.11 ± 0.48	3.88 ± 0.48	3.57 ± 0.52	3.82 ± 0.30	3.84 ± 0.25
ESD, mm	2.47 ± 0.58	2.82 ± 0.47	2.60 ± 0.44	2.51 ± 0.37	2.62 ± 0.27	2.46 ± 0.44
EF, %	74.13 ± 8.26	66.51 ± 7.85	68.18 ± 6.41	65.66 ± 6.34	67.08 ± 0.18	71.25 ± 9.28
FS, %	38.20 ± 8.10	32.25 ± 5.38	33.00 ± 4.70	31.00 ± 4.41	31.96 ± 3.76	36.18 ± 8.83
**Weight, g**	23.65 ± 2.50	26.45 ± 2.87	28.08 ± 2.82	39.06 ±2.74^*^	45.28 ±3.02^*‡^	50.98 ±2.49^*‡^
**Isolated heart (n= 5 for wild-type; n= 7 for db/db)**						
LVW, g			0.12 ± 0.03			0.13 ± 0.03
HW, g			0.15 ± 0.03			0.16 ±0.03
HW/BW (%)			0.60 ± 0.01^†^			0.31 ± 0.06

LVW, left ventricular weight. HW, heart weight. BW, body weight. UCG, ultrasound cardiogram. IVS, interventricular septum.

PW, posterior wall, EDD, end diastolic dimension. ESD, end systolic dimension. EF, ejection fraction. FS, fractional shortening.

^*^Significantly higher than wild-type group.

^†^Significantly higher than db/db group.

^‡^Significantly higher than baseline W8 values.

P<0.05 was considered significant.

**Figure 2:**
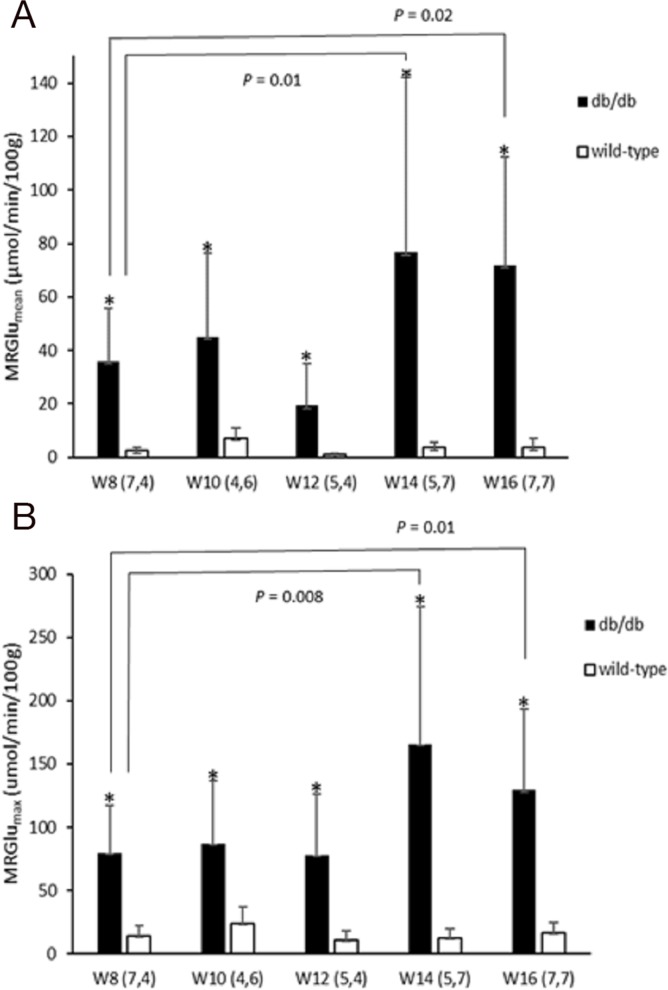
Metabolic glucose utilization of the myocardium [MRGlu_mean_ **(A)** and MRGlu_max_ **(B)**] in wild type and db/db mice at 8, 10, 12, 14, and 16 weeks of age. Significantly higher MRGlu values were observed in db/db mice as compared with age-matched controls (all *P*<0.05). A statistically significant trend was also seen with time. Significance values are denoted above bar plots.

**Figure 3:**
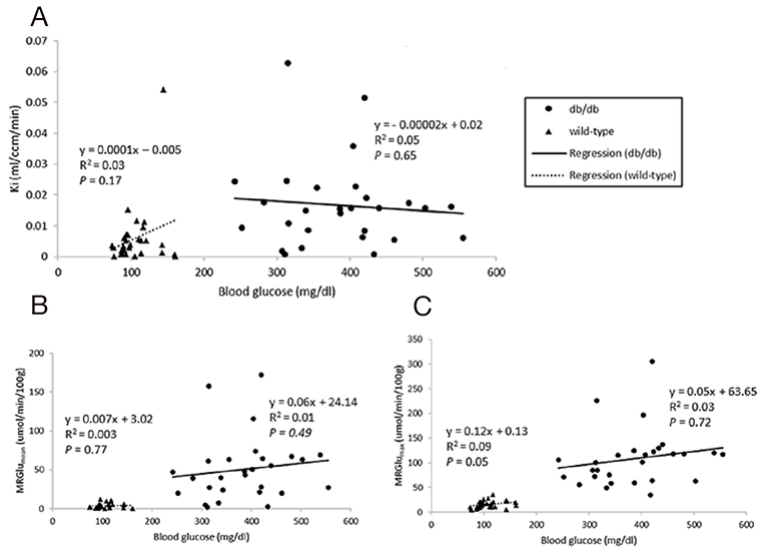
Relationships between blood glucose levels and Ki **(A)**, MRGlu_mean_ **(B)**, MRGlu_max_ **(C)** in the myocardium. *P* value of less than 0.05 was considered significant.

**Figure 4:**
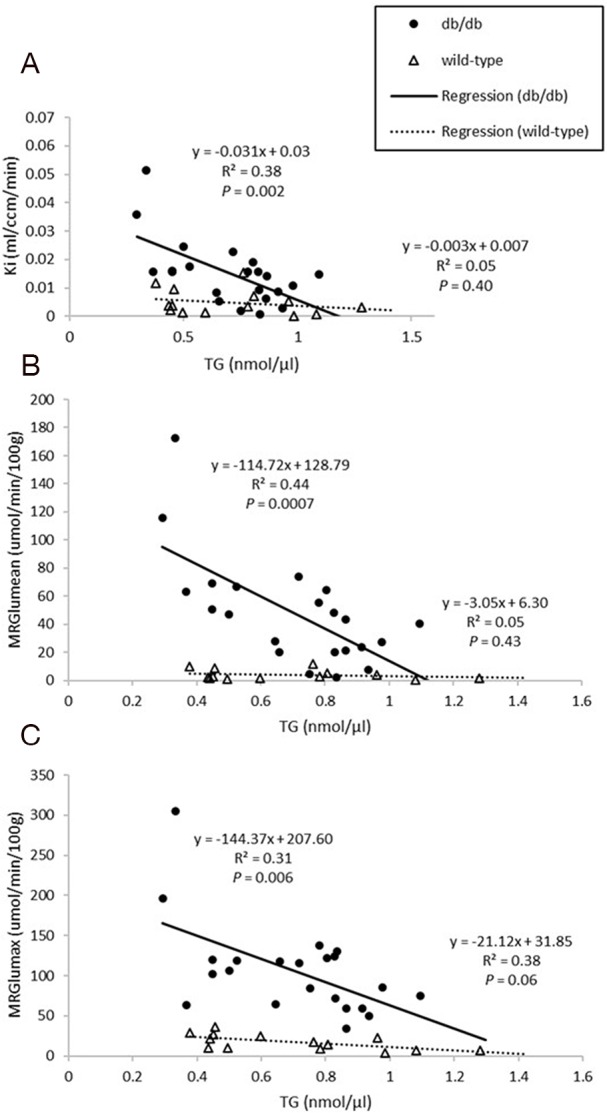
Relationships between plasma triglyceride (TG) levels and Ki **(A)**, MRGlu_mean_ **(B)**, MRGlu_max_ **(C)** in the myocardium. Ki and MRGlu were both inversely proportional to TG in db/db mice (all *P*<0.05).

Dynamic PET parameters are depicted in Table 1. The db/db mice had significantly higher glucose uptake rates (MRGlu_mean_, MRGlu_max_) compared with age-matched controls (Table 1, Figure 2). At W12, either db/db mice or wild-type mice tended to have higher TG as compared with other time points which might resulted relatively lower MRGlu. Although the variability in maximum and mean values of MRGlu was observed, similar trends of myocardial glucose uptake rates described above existed in all-time points. In db/db mice, there were mild correlations between age and MRGlu_mean_ (R^2^ = 0.142, *P* = 0.047), or MRGlu_max_ (R^2^ = 0.198, *P* = 0.017), and MRGlu values were significantly higher than baseline after age of 14 week. Regarding the relationship between K_i_, MRGlu, and glucose level, no significant correlation was observed in neither db/db nor wild-type mice group (Figure 3). When both groups were pooled together, K_i_ (R^2^ = 0.13, *P* = 0.004) and MRGlu (R^2^ = 0.38, *P* < 0.0001 for MRGlu_mean_; R^2^ = 0.55, *P* < 0.0001 for MRGlu_max_) depended on glucose level modestly. On the other hand, in db/db mice, we observed that K_i_ (R^2^= 0.38, *P* = 0.002) and MRGlu (R^2^= 0.44, *P* = 0.0007 for MRGlu_mean_; R^2^= 0.31, *P* = 0.006 for MRGlu_max_) were both inversely proportional to TG (Figure 4).

## DISCUSSION

Fatty acid oxidation is the predominant source (60-90%) of adenosine triphosphate in the normal heart, with glucose oxidation and lactate contributing the rest [25]. In the diabetic heart, the contribution of glucose to total overall energy expenditure is diminished such that the heart muscle relies almost entirely on fatty acids for energy [26]. Ours is the first study to address the early metabolic alterations of LV myocardium in the obesity, type 2 DM mouse model using small animal PET/CT. Based on our findings, metabolic changes appear to precede morphological and mechanical changes of LV related to circulating substrate shifts.

PET imaging of the heart has been used to evaluate the myocardial metabolic pathways in diabetes in animal models as well as humans [13, 27–33]. In the present study, we demonstrated that before development of LV hypertrophy, db/db mice with preserved LV systolic function had enhanced myocardial glucose utilization in the early stage of type 2 diabetes and obesity, associated with circulating substrates shifting. Contradictory to our study, previous small animal studies have demonstrated reduced myocardial glucose uptake or utilization in the diabetic heart, suggesting abnormalities in glucose transport. In four dynamic animal PET studies (Table 4), designs of the diabetic animal model, animal age and time points of PET imaging were different from those in our experiment. Two did not provide LV function data. On the other hand, two db/db mice studies [27, 31] reported diminished myocardial glucose uptake. Yue et al. [27] showed that LV mass and wall thickness were significantly increased in db/db mice group. Abdurrachim et al. [31] found diabetic mice had normal cardiac function, higher myocardial TG (~50%) and lower ^18^F-FDG uptake (44%), and which the values of glucose uptake were seemed to be normalized upon pressure overload. The major difference was static ^18^F-FDG PET with semi-quantitative method. MRGlu provided a more accurate evaluation of glucose consumption as compared with semi-quantitative method [34]. In addition, baseline characteristics including age, circulating substrate levels other than plasma glucose, and control group (wild-type vs. heterozygous control), were different from our study, which might influence the myocardial glucose utilization. In human research, Kim et al. [32] found that myocardial glucose uptake on static ^18^F-FDG PET was significantly decreased in subjects with type 2 diabetes compared to the normal glucose tolerance or prediabetes groups. On the other hand, one quantitative study with ^18^F-FDG under hyperinsulinemic-euglycemic clamping demonstrated that young male patients with less than a five-year history of insulin-dependent diabetes had similar insulin-stimulated myocardial glucose uptake compared with healthy controls [33]. The inconsistent findings might be related to study design, methodologies and most human studies on ^18^F-FDG PET entailing a long history of type 2 DM plus multiple comorbidities and heterogeneous medications which these factors confound assessment of myocardial glucose utilization.

**Table 3:** Experimental designs

Mice models	Experiments	Time point (age in week)
1. db/db vs. wild-type (8/group)	NPOFDG PET (MRGlu)HOMA-IR, glucose, TG, FFA	8, 10, 12, 14, 16
2. db/db vs. wild-type (8/group)	Echocardiography	8, 12, 16

MRGlu, rate of myocardial glucose uptake. NPO, nil per os. HOMA-IR, homeostasis model assessment-estimated insulin resistance.

TG, triglyceride. FFA, free fatty acid.

**Table 4:** Previous FDG PET studies of animal heart *in vivo* for assessment of myocardial glucose utilization

Reference	Animal models	FDG PET	Time points	Injection route	Glucose clamp	glucose uptake/MRGlu	LV function
Yue et al., 2007 [27]	db/db and db/+ mice	Static	W9	IV	Insulin tolerance test	↓glucose uptake	Preserved EF
Shoghi et al., 2008 [13]	Type 2 diabetic ZDF rat	Dynamic	W14, W19	IV	No	↓MRGlu, no difference in aging	Preserved FS
Ménard et al., 2010 [28]	Diet-induced Type 2 diabetic rat	Dynamic	W14	IV	Yes	↓MRGlu	↓EF
Thorn et al., 2013 [29]	Type 1 diabetic STZ mice	Dynamic	Baseline, 2wk after STZ	IV	No	↓MRGlu	No mention
Nemanich et al., 2013 [30]	Type 2 diabetic ZDF rat	Dynamic	W14	IV	No	↓MRGlu	No mention
Abdurrachim et al., 2017 [31]	db/db and db/+ mice	Static	W10	IV	No	↓glucose uptake	Preserved EF

ZDF, Zucker Diabetic Fatty. STZ, streptozotocin. IV, intravenous. MRGlu, rate of myocardial glucose uptake. FS, fractional shortening. EF, ejection fraction.

↑and↓stand for increased and decreased effect, respectively.

The natural history of db/db mice exhibited a distinct pattern [35, 36]. In the beginning, peripheral insulin resistance could be overcome by increased insulin secretion. Hyperglycemia developed when enhanced insulin secretion could no longer compensate for insulin resistance. Body weights of db/db mice increase progressively with greater lipolysis of endogenous adipose stores, leading to increased levels of circulating FFAs. Concordant to our findings, obesity, hyperglycemia, and elevated HOMA-IR index were present in db/db mice. The former two factors became progressively obvious as aging, in comparison with their genetic controls. Additionally, db/db mice tended to have higher circulating FFA and insulin levels, albeit non-significantly as compared with wild-type mice.

The most important finding of this study was that db/db mice had a progressively increased rate of LV myocardial glucose uptake compared with wild-type mice, without significant changes on LV volumetric data, systolic function and mass, till 16 weeks of age. ^18^F-FDG myocardial uptake varies greatly, because cardiac myocytes can derive energy from various competitive substrates, including FA, glucose, lactate, pyruvate, and ketones, depending on various physiologic and pathologic conditions. Glucose uptake is dependent on the transmembrane glucose gradient and density of glucose transporters and increased circulating glucose concentration and decreased FA levels could enhance glucose uptake [13, 28, 37]. Our data pointed out the trends that K_i_ and myocardial MRGlu values correlated with glucose concentration positively and with TG levels negatively. Taken together, it was speculated that early metabolic remodeling, which could be considered as adaptive, might precede the onset of contractile dysfunction in DM. Interestingly, Zhong et al. [15] have found a similar early adaptive response, enhanced myocardial glucose uptake, in a mouse model of pressure overload LV hypertrophy *in vivo*. Longitudinal evaluation of myocardial glucose metabolism and contractile function using dynamic ^18^F-FDG PET and echocardiography allow us to track the footprints of deranged metabolic activity, LV structure and function in the diabetic heart. Although the mechanistic details leading to diabetic cardiomyopathy remain unclear, restoring cardiac metabolism seem to be a cornerstone. In light of the data provided here, a prospective therapeutic strategy focused on correcting the early imbalance of cardiac metabolism may be outlined and may prevent cardiac malfunction and lethal coronary events. Additionally, the emergence of new heart-specific drugs for type 2 DM that target specific metabolic processes is creating a need to direct and monitor metabolic response. This is paving a path for personalized therapy directed by metabolic imaging [38].

Several issues should be considered when interpreting our results. Although db/db mice or wild-type mice had the relatively lower MRGlu at W12 which might be resulted from increased TG, the trend of myocardial glucose uptake rate described above existed in all-time points. The transient cause of plasma TG elevation was not clearly understood, possibly related to dietary condition or fasting hours. The significant correlation between glucose level and myocardial glucose uptake rate was only seen in pooled data. The reason might be due to that plasma glucose levels were significantly different between db/db and wild-type groups, while the range of plasma glucose levels within group was rather small, thus the statistical power was not enough in small sample size of each group.

Dietary conditions affect ^18^F-FDG PET imaging and it has been known that myocardial glucose uptake is diminished by fasting [39, 40]. Although some studies have used glucose load conditioning, fasting could make metabolic conditions uniform and produce similar imaging quality [15]. Because our purpose was to determine the rate of myocardial glucose uptake, a reasonable contrast between LV cavity and myocardial tissue was enough for us to draw regions of interest. Hyperinsulinemic-euglycaemic clamp was regarded as the gold standard in human ^18^F-FDG PET imaging. However, it was difficult to adopt in the rodent experiments (Table 4). Because glucose levels may affect myocardial glucose utilization, we performed HOMA-IR and multiple regression analysis to evaluate the effects of circulating substrate levels on the rate of myocardial glucose uptake. However, we did not validate our results based on circulating substrate analysis, echocardiography, and PET measurement of glucose utilization by correlation to histological, tissue substrate, genetic or protein expression studies.

There were some methodologic limitations. We utilized intraperitoneal ^18^F-FDG injection instead of tail-vein injection. Although tail-vein injection is probably the route of choice for administrating radiopharmaceuticals or drugs to rodents in dynamic study, it is not easy to perform on mice. Additionally, reproducible intravenous injection is not possible for longitudinal studies. Intraperitoneal injection appears to be an alternative and has been proven to be reproducible, thereby reducing stress to the animal [41, 42]. Wong et al. 2011 [39] reported that both intraperitoneal and tail-vein routes of administrations provided equivalent pharmacokinetic parameters and comparable ^18^F-FDG biodistribution within 60 minutes after injection. Invasive nature and limited blood volume in mice precludes repeated blood sampling rendering imaged-derived input function desirable for quantification of glucose uptake. In our study, the LV cavity was used to measure ^18^F-FDG input function. However, in small animals, this image-derived volume might be less effective because limited spatial resolution and partial-volume effects result in spill-over from the myocardial tissue in later frames [28, 29, 43]. In our study, 17.5 % of PET images were uninterpretable, mostly because of non-, scarce or heterogeneous myocardial ^18^F-FDG uptake. Although several methods measuring the input function, such as a hybrid modeling approach [44] and an inferior vena cava time-activity curve [45, 46], have been proposed, further validations should be undertaken. The LC used to account for differences in transport and phosphorylation rates between ^18^F-FDG and glucose is an important parameter in the determination of MRGlu in the dynamic PET [47, 48]. However, this value may vary, depending on circulating substrate and hormonal conditions. In the current work, the constant applied (LC = 0.67) was originally suggested for rabbits and modified for mice and rats [29, 46, 49]. The LC proportionally affected the precision of the MRGlu value, but not the relative difference between the two groups. PET allows simultaneous measurements of radiotracer uptake and tissue kinetics in multiple organs [50–52]. However, we did not compare skeletal muscle due to higher background activity after intraperitoneal ^18^F-FDG injection.

For reducing the physical and psychological stress from the anesthesia procedure, we designed two experimental models instead of one group receiving both PET study and echocardiographic measurement. Therefore, the animals were not exactly the same in two experiments. Moreover, due to unexpected death from the anesthesia procedure in mice, experiments could not be performed on all mice. Further work using finite element mechanical model to determine ventricular deformation directly from nuclear imaging data will be considered [53].

Finally, isoflurane anesthesia is known to affect myocardial glucose uptake [54, 55]. However, this limitation is unlikely to influence the inter-group comparison, because all animals were exposed to the same anesthesia.

## MATERIALS AND METHODS

### Animals used in this study

All experiments were performed in compliance with experimental animal care guidelines, and were conducted under protocols approved by the Institutional Animal Care and Use Committee at National Taiwan University College of Medicine and Far Eastern Memorial Hospital. Adult male db/db mice (BKS.Cg- Dock 7m +/+ Leprdb/JNarl) and wild-type C57BL mice (C57BL/6JNarl, used as non-diabetic controls) were obtained from the Laboratory Animal Center at National Taiwan University College of Medicine (Taipei, Taiwan). Experiments began when animals were 8 weeks of age. As shown in Table 3, in order to reduce the stress to animals and death from the anesthesia procedure, two protocols for PET and echocardiography were designed [27, 56, 57]. Sixteen mice (8 db/db, 8 control) underwent PET imaging and plasma subject analysis, including homeostasis model assessment-estimated insulin resistance (HOMA-IR), in 2-week intervals. A second group of eight db/db mice and 8 control (wild type) mice received echocardiography in 4-week interval.

### Animal preparation

The animals were freely fed standard laboratory diet and water in temperature-controlled facilities with a 12-hour light, 12-hour dark cycle (lights on at 6:00AM). Only before PET imaging, food was taken away at least 12 hours while water was given *ad libitum* in the evening before the studies. For PET and echocardiographic studies, mice were anesthetized via inhalational isoflurane and medical-grade oxygen. The gases were administered, via an induction chamber and custom-designed nose cone, at 2-2.5% isoflurane for induction and 1-1.5% isoflurane for maintenance throughout the imaging session. After completion of all experiments, anaesthetized animals were sacrificed and the heart was excised and weighed.

### Small-animal PET imaging protocol and imaging analysis

**Figure 5:**
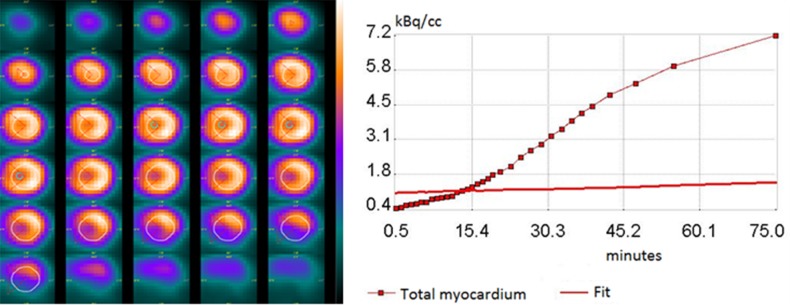
Representative of myocardial time-activity curve together with the ^18^ F-FDG kinetics fitted model curve (right), and its transverse PET images in fasting mice after intraperitoneal injection of ^18^ F-FDG (left) The highlighted circle and radius demonstrated the circular volume-of-interest (VOI) which has a 60° angle indicating the septum location.

Small-animal PET was performed on the Argus PET-CT scanner (SEDECAL, Madrid, Spain). Prior to administration of ^18^F-FDG, each mouse received a scout scan for localization. Dynamic PET imaging was started immediately after ^18^F-FDG (15.0 ± 3.0 MBq) injection into the peritoneal cavity and continued for 90 minutes (20 × 60, 10 × 120, 2 × 300, 1 × 600, 1 × 1,800-s) in prone position [39]. After the PET scan, a CT study was acquired and the list-mode PET data were reconstructed using a filtered back projection algorithm with CT-based attenuation correction. Images were analyzed in a blinded manner using PMOD 3.6 software (PMOD Technologies Ltd., Zürich, Switzerland), which provides the quantification of dynamic PET of the heart with a broad range of tracers in small animals including rodents [46, 58, 59]. In brief, volumes of interest (VOI) were drawn semi-automatically by use of a center-line within the myocardium in the projection of three dimensions (short-axis, vertical long, horizontal-long). To generate blood pool time-activity curves, VOI were placed within the LV cavity with care taken to avoid the boundary of myocardium and LV cavity and to reduce the partial volume effect. Time-activity curves were fitted to a Patlak kinetic model (Figure 5). The rate of myocardial glucose uptake (MRGlu) was calculated as MRGlu = KiCglu/LC, where Ki is the graphically defined Patlak slope, Cglu is the mean plasma glucose concentration at the start and end of the scan, and LC is the lumped constant, which is assumed to be 0.67 as estimated for rodents. The data of MRGlu were summarized by volume-weighting averaging to a 17-segment model based on the American Heart Association classification. To exclude the potentially cofounding effects from averaging multiple segments (MRGlu_mean_), values in the maximal segment of each subject (MRGlu_max_) were also calculated.

### Circulating substrate analysis and HOMA-IR Index

On the day of the PET study, whole blood samples were drawn from the retro-orbital venous sinus for substrate analysis. Plasma glucose levels were measured by placing whole blood on a glucose test strip for immediate analysis, using a plasma blood glucose analyzer [Accu-Chek; Roche Diagnostics, Inc.]). Serum samples were separated from whole blood samples by centrifuging at 13,000 rpm for 20 min (Axygen® 1.5mL MaxyClear Snaplock Microcentrifuge Tube, Polypropylene, Red, Nonsterile, Product #MCT-150-R) and placed in a −20°C freezer prior to analysis. Triglyceride (TG) and free fatty acid (FFA) levels were measured using Quantification Colorimetric/Fluorometric Kits (BioVision, CA, USA). Insulin levels were measured using a mouse insulin ELISA test kit (Mercodia, Uppsala, Sweden). All kits were commercially available, and used well-documented methods that have been validated in small animals [60], and all measurements had an SD of ≤5%.

HOMA-IR, developed by Matthews *et al*. [61] has been used widely in research for estimation of insulin resistance. The value was calculated by multiplying fasting plasma insulin (mU/L) by fasting plasma glucose (mmol/L), then dividing by the constant 22.5. The substrate values reported in the study correspond to values obtained at the baseline of PET, just before ^18^F-FDG injection.

### Echocardiography measurement

Echocardiography on mice was performed using a GE Vivid 7 Ultrasound machine (General Electric Company, Fairfield, CT, USA) with GE I13L probe (5.8 to 14.0 MHz) at ages 8, 12, and 16 weeks. Animals were anesthetized via isoflurane inhalation and secured on an imaging platform in the supine position, then the anterior chest was shaved and ultrasonic coupling gel was applied. Care was taken to maintain adequate contact and to avoid excessive pressure on the chest. Complete 2-dimensional and M-mode examinations were performed from multiple views. Mean fractional shortening (FS), left ventricular ejection fraction (LVEF), end-diastolic dimension (EDD), and end-systolic dimension (ESD) were obtained after repeated measurement for three times by two operators (Liu CW, Wu YW) in consensus [62].

### Statistics

Data processing and statistical analysis were performed using either Microsoft Excel or Medcalc Statistical software (Brunswick, Maine, USA). Continuous variables were expressed as mean±SD. The two-tailed student t-test or Mann-Whitney rank sum test were performed to test for significant difference between and within groups. Regression analysis was performed in the two groups to characterize the dependence of blood glucose, body weight, MRGlu on age and Ki, MRGlu on circulating substrate levels. The significance level was set at 0.05.

Regarding sample size, power analysis (type 1 error: 5%; power: 80%; two-tailed test; t-test for difference of myocardial glucose utilization between control and diabetic mice) and the information of effect size was taken from previously studies [13, 16]. The result was 7 animals per group with expecting 10% attrition, and final sample size 8 animals per group.

## CONCLUSION

In our study, noninvasive serial imaging of the diabetic heart using dynamic PET has revealed that enhanced uptake rate of ^18^F-FDG by the myocardium in type 2 DM and obese mice precedes the onset of LV hypertrophy and contractile dysfunction.

## References

[1] Fang ZY, Prins JB, Marwick TH (2004). Diabetic cardiomyopathy: evidence, mechanisms, and therapeutic implications. Endocr Rev.

[2] Boudina S, Abel ED (2010). Diabetic cardiomyopathy, causes and effects. Rev Endocr Metab Disord.

[3] Lopaschuk GD, Belke DD, Gamble J, Itoi T, Schonekess BO (1994). Regulation of fatty acid oxidation in the mammalian heart in health and disease. Biochim Biophys Acta.

[4] Lopaschuk GD (2002). Metabolic abnormalities in the diabetic heart. Heart Fail Rev.

[5] Garvey WT, Hardin D, Juhaszova M, Dominguez JH (1993). Effects of diabetes on myocardial glucose transport system in rats: implications for diabetic cardiomyopathy. Am J Physiol.

[6] Yamaji T, Fukuhara T, Kinoshita M (1993). Increased capillary permeability to albumin in diabetic rat myocardium. Circ Res.

[7] Taylor M, Wallhaus TR, Degrado TR, Russell DC, Stanko P, Nickles RJ, Stone CK (2001). An evaluation of myocardial fatty acid and glucose uptake using PET with [18F]fluoro-6-thia-heptadecanoic acid and [18F]FDG in Patients with Congestive Heart Failure. J Nucl Med.

[8] Maki MT, Haaparanta M, Nuutila P, Oikonen V, Luotolahti M, Eskola O, Knuuti JM (1998). Free fatty acid uptake in the myocardium and skeletal muscle using fluorine-18-fluoro-6-thia-heptadecanoic acid. J Nucl Med.

[9] DeGrado TR, Coenen HH, Stocklin G (1991). 14(R, S)-[18F]fluoro-6-thia-heptadecanoic acid (FTHA): evaluation in mouse of a new probe of myocardial utilization of long chain fatty acids. J Nucl Med.

[10] Visser EP, Disselhorst JA, Brom M, Laverman P, Gotthardt M, Oyen WJ, Boerman OC (2009). Spatial resolution and sensitivity of the Inveon small-animal PET scanner. J Nucl Med.

[11] Shoghi KI (2009). Quantitative small animal PET. Q J Nucl Med Mol Imaging.

[12] Yamane T, Park MJ, Richter D, Nekolla SG, Javadi MS, Lapa C, Samnick S, Buck AK, Herrmann K, Higuchi T (2014). Small-animal PET imaging of isolated perfused rat heart. J Nucl Med.

[13] Shoghi KI, Gropler RJ, Sharp T, Herrero P, Fettig N, Su Y, Mitra MS, Kovacs A, Finck BN, Welch MJ (2008). Time course of alterations in myocardial glucose utilization in the Zucker diabetic fatty rat with correlation to gene expression of glucose transporters: a small-animal PET investigation. J Nucl Med.

[14] Devanathan S, Nemanich ST, Kovacs A, Fettig N, Gropler RJ, Shoghi KI (2013). Genomic and metabolic disposition of non-obese type 2 diabetic rats to increased myocardial fatty acid metabolism. PLoS One.

[15] Zhong M, Alonso CE, Taegtmeyer H, Kundu BK (2013). Quantitative PET imaging detects early metabolic remodeling in a mouse model of pressure-overload left ventricular hypertrophy in vivo. J Nucl Med.

[16] Hernandez AM, Huber JS, Murphy ST, Janabi M, Zeng GL, Brennan KM, O’Neil JP, Seo Y, Gullberg GT (2013). Longitudinal evaluation of left ventricular substrate metabolism, perfusion, and dysfunction in the spontaneously hypertensive rat model of hypertrophy using small-animal PET/CT imaging. J Nucl Med.

[17] Chen H, Charlat O, Tartaglia LA, Woolf EA, Weng X, Ellis SJ, Lakey ND, Culpepper J, Moore KJ, Breitbart RE, Duyk GM, Tepper RI, Morgenstern JP (1996). Evidence that the diabetes gene encodes the leptin receptor: identification of a mutation in the leptin receptor gene in db/db mice. Cell.

[18] Hummel KP, Dickie MM, Coleman DL (1966). Diabetes, a new mutation in the mouse. Science.

[19] Robinson R, Barathi VA, Chaurasia SS, Wong TY, Kern TS (2012). Update on animal models of diabetic retinopathy: from molecular approaches to mice and higher mammals. Dis Model Mech.

[20] Li J, Wang JJ, Yu Q, Chen K, Mahadev K, Zhang SX (2010). Inhibition of reactive oxygen species by Lovastatin downregulates vascular endothelial growth factor expression and ameliorates blood-retinal barrier breakdown in db/db mice: role of NADPH oxidase 4. Diabetes.

[21] Kobayashi K, Forte TM, Taniguchi S, Ishida BY, Oka K, Chan L (2000). The db/db mouse, a model for diabetic dyslipidemia: molecular characterization and effects of Western diet feeding. Metabolism.

[22] Semeniuk LM, Kryski AJ, Severson DL (2002). Echocardiographic assessment of cardiac function in diabetic db/db and transgenic db/db-hGLUT4 mice. Am J Physiol Heart Circ Physiol.

[23] Belke DD, Larsen TS, Gibbs EM, Severson DL (2000). Altered metabolism causes cardiac dysfunction in perfused hearts from diabetic (db/db) mice. Am J Physiol Endocrinol Metab.

[24] Buchanan J, Mazumder PK, Hu P, Chakrabarti G, Roberts MW, Yun UJ, Cooksey RC, Litwin SE, Abel ED (2005). Reduced cardiac efficiency and altered substrate metabolism precedes the onset of hyperglycemia and contractile dysfunction in two mouse models of insulin resistance and obesity. Endocrinology.

[25] Taegtmeyer H, Hems R, Krebs HA (1980). Utilization of energy-providing substrates in the isolated working rat heart. Biochem J.

[26] Saddik M, Lopaschuk GD (1994). Triacylglycerol turnover in isolated working hearts of acutely diabetic rats. Can J Physiol Pharmacol.

[27] Yue P, Arai T, Terashima M, Sheikh AY, Cao F, Charo D, Hoyt G, Robbins RC, Ashley EA, Wu J, Yang PC, Tsao PS (2007). Magnetic resonance imaging of progressive cardiomyopathic changes in the db/db mouse. Am J Physiol Heart Circ Physiol.

[28] Menard SL, Croteau E, Sarrhini O, Gelinas R, Brassard P, Ouellet R, Bentourkia M, van Lier JE, Des Rosiers C, Lecomte R, Carpentier AC (2010). Abnormal in vivo myocardial energy substrate uptake in diet-induced type 2 diabetic cardiomyopathy in rats. Am J Physiol Endocrinol Metab.

[29] Thorn SL, deKemp RA, Dumouchel T, Klein R, Renaud JM, Wells RG, Gollob MH, Beanlands RS, DaSilva JN (2013). Repeatable noninvasive measurement of mouse myocardial glucose uptake with 18F-FDG: evaluation of tracer kinetics in a type 1 diabetes model. J Nucl Med.

[30] Nemanich S, Rani S, Shoghi K (2013). *In vivo* multi-tissue efficacy of peroxisome proliferator-activated receptor-gamma therapy on glucose and fatty acid metabolism in obese type 2 diabetic rats. Obesity (Silver Spring).

[31] Abdurrachim D, Nabben M, Hoerr V, Kuhlmann MT, Bovenkamp P, Ciapaite J, Geraets IM, Coumans W, Luiken J, Glatz JF, Schafers M, Nicolay K, Faber C (2017). Diabetic db/db mice do not develop heart failure upon pressure overload: a longitudinal in vivo PET, MRI, and MRS study on cardiac metabolic, structural, and functional adaptations. Cardiovasc Res.

[32] Kim G, Jo K, Kim KJ, Lee YH, Han E, Yoon HJ, Wang HJ, Kang ES, Yun M (2015). Visceral adiposity is associated with altered myocardial glucose uptake measured by (18)FDG-PET in 346 subjects with normal glucose tolerance, prediabetes, and type 2 diabetes. Cardiovasc Diabetol.

[33] vom Dahl J, Herman WH, Hicks RJ, Ortiz-Alonso FJ, Lee KS, Allman KC, Wolfe ER, Kalff V, Schwaiger M (1993). Myocardial glucose uptake in patients with insulin-dependent diabetes mellitus assessed quantitatively by dynamic positron emission tomography. Circulation.

[34] Dilsizian V, Bacharach SL, Beanlands RS, Bergmann SR, Delbeke D, Dorbala S, Gropler RJ, Knuuti J, Schelbert HR, Travin MI (2016). ASNC imaging guidelines/SNMMI procedure standard for positron emission tomography (PET) nuclear cardiology procedures. J Nucl Cardiol.

[35] Aasum E, Hafstad AD, Severson DL, Larsen TS (2003). Age-dependent changes in metabolism, contractile function, and ischemic sensitivity in hearts from db/db mice. Diabetes.

[36] Aasum E, Belke DD, Severson DL, Riemersma RA, Cooper M, Andreassen M, Larsen TS (2002). Cardiac function and metabolism in Type 2 diabetic mice after treatment with BM 17.0744, a novel PPAR-alpha activator. Am J Physiol Heart Circ Physiol.

[37] Menard SL, Ci X, Frisch F, Normand-Lauziere F, Cadorette J, Ouellet R, Van Lier JE, Benard F, Bentourkia M, Lecomte R, Carpentier AC (2009). Mechanism of reduced myocardial glucose utilization during acute hypertriglyceridemia in rats. Mol Imaging Biol.

[38] Fuentes-Antras J, Picatoste B, Ramirez E, Egido J, Tunon J, Lorenzo O (2015). Targeting metabolic disturbance in the diabetic heart. Cardiovasc Diabetol.

[39] Wong KP, Sha W, Zhang X, Huang SC (2011). Effects of administration route, dietary condition, and blood glucose level on kinetics and uptake of 18F-FDG in mice. J Nucl Med.

[40] Opie LH, Evans JR, Shipp JC (1963). Effect of fasting on glucose and palmitate metabolism of perfused rat heart. Am J Physiol.

[41] Marsteller DA, Barbarich-Marsteller NC, Fowler JS, Schiffer WK, Alexoff DL, Rubins DJ, Dewey SL (2006). Reproducibility of intraperitoneal 2-deoxy-2-[18F]-fluoro-D-glucose cerebral uptake in rodents through time. Nucl Med Biol.

[42] Schiffer WK, Mirrione MM, Dewey SL (2007). Optimizing experimental protocols for quantitative behavioral imaging with 18F-FDG in rodents. J Nucl Med.

[43] Shoghi KI, Welch MJ (2007). Hybrid image and blood sampling input function for quantification of small animal dynamic PET data. Nucl Med Biol.

[44] Wong KP, Zhang X, Huang SC (2013). Improved derivation of input function in dynamic mouse [18F]FDG PET using bladder radioactivity kinetics. Mol Imaging Biol.

[45] Lanz B, Poitry-Yamate C, Gruetter R (2014). Image-derived input function from the vena cava for 18F-FDG PET studies in rats and mice. J Nucl Med.

[46] Thackeray JT, Bankstahl JP, Bengel FM (2015). Impact of image-derived input function and fit time intervals on patlak quantification of myocardial glucose uptake in mice. J Nucl Med.

[47] Huang SC, Phelps ME, Hoffman EJ, Sideris K, Selin CJ, Kuhl DE (1980). Noninvasive determination of local cerebral metabolic rate of glucose in man. Am J Physiol.

[48] Sokoloff L, Reivich M, Kennedy C, Des Rosiers MH, Patlak CS, Pettigrew KD, Sakurada O, Shinohara M (1977). The [14C]deoxyglucose method for the measurement of local cerebral glucose utilization: theory, procedure, and normal values in the conscious and anesthetized albino rat. J Neurochem.

[49] Krivokapich J, Huang SC, Selin CE, Phelps ME (1987). Fluorodeoxyglucose rate constants, lumped constant, and glucose metabolic rate in rabbit heart. Am J Physiol.

[50] Cochran BJ, Ryder WJ, Parmar A, Tang S, Reilhac A, Arthur A, Charil A, Hamze H, Barter PJ, Kritharides L, Meikle SR, Gregoire MC, Rye KA (2016). In vivo PET imaging with [(18)F]FDG to explain improved glucose uptake in an apolipoprotein A-I treated mouse model of diabetes. Diabetologia.

[51] Mathew D, Zhou P, Pywell CM, van der Veen DR, Shao J, Xi Y, Bonar NA, Hummel AD, Chapman S, Leevy WM, Duffield GE (2013). Ablation of the ID2 gene results in altered circadian feeding behavior, and sex-specific enhancement of insulin sensitivity and elevated glucose uptake in skeletal muscle and brown adipose tissue. PLoS One.

[52] Kreissl MC, Stout DB, Wong KP, Wu HM, Caglayan E, Ladno W, Zhang X, Prior JO, Reiners C, Huang SC, Schelbert HR (2011). Influence of dietary state and insulin on myocardial, skeletal muscle and brain [F]-fluorodeoxyglucose kinetics in mice. EJNMMI Res.

[53] Veress AI, Weiss JA, Huesman RH, Reutter BW, Taylor SE, Sitek A, Feng B, Yang Y, Gullberg GT (2008). Measuring regional changes in the diastolic deformation of the left ventricle of SHR rats using microPET technology and hyperelastic warping. Ann Biomed Eng.

[54] Toyama H, Ichise M, Liow JS, Vines DC, Seneca NM, Modell KJ, Seidel J, Green MV, Innis RB (2004). Evaluation of anesthesia effects on [18F]FDG uptake in mouse brain and heart using small animal PET. Nucl Med Biol.

[55] Fueger BJ, Czernin J, Hildebrandt I, Tran C, Halpern BS, Stout D, Phelps ME, Weber WA (2006). Impact of animal handling on the results of 18F-FDG PET studies in mice. J Nucl Med.

[56] Barouch LA, Berkowitz DE, Harrison RW, O’Donnell CP, Hare JM (2003). Disruption of leptin signaling contributes to cardiac hypertrophy independently of body weight in mice. Circulation.

[57] Dludla PV, Essop MF, Gabuza KB, Muller CJF, Louw J, Johnson R (2017). Age-dependent development of left ventricular wall thickness in type 2 diabetic (db/db) mice is associated with elevated low-density lipoprotein and triglyceride serum levels. Heart Vessels.

[58] Choi H, Han JH, Lim SY, Lee I, Cho YS, Chun EJ, Lee WW (2017). Imaging of myocardial ischemia-reperfusion injury using sodium [18F]fluoride positron emission Tomography/Computed Tomography in Rats and Humans. Mol Imaging.

[59] Clemmensen AE, Ghotbi AA, Bodholdt RP, Hag AMF, Hasbak P, Ripa RS, Kjaer A (2017). Perfusion imaging using rubidium-82 (82Rb) PET in rats with myocardial infarction: first small animal cardiac 82Rb-PET. J Nucl Cardiol.

[60] Sharp TL, Dence CS, Engelbach JA, Herrero P, Gropler RJ, Welch MJ (2005). Techniques necessary for multiple tracer quantitative small-animal imaging studies. Nucl Med Biol.

[61] Matthews DR, Hosker JP, Rudenski AS, Naylor BA, Treacher DF, Turner RC (1985). Homeostasis model assessment: insulin resistance and beta-cell function from fasting plasma glucose and insulin concentrations in man. Diabetologia.

[62] Liu YH, Peng KY, Chiu YW, Ho YL, Wang YH, Shun CT, Huang SY, Lin YS, de Vries AA, Pijnappels DA, Lee NT, Yen BL, Yen ML (2015). Human placenta-derived multipotent cells (hPDMCs) modulate cardiac injury: from bench to small and large animal myocardial ischemia studies. Cell Transplant.

